# Variation in RNA Virus Mutation Rates across Host Cells

**DOI:** 10.1371/journal.ppat.1003855

**Published:** 2014-01-23

**Authors:** Marine Combe, Rafael Sanjuán

**Affiliations:** 1 Instituto Cavanilles de Biodiversidad y Biología Evolutiva, Valencia, Spain; 2 Departament de Genètica, Universitat de València, Valencia, Spain; University of Michigan, United States of America

## Abstract

It is well established that RNA viruses exhibit higher rates of spontaneous mutation than DNA viruses and microorganisms. However, their mutation rates vary amply, from 10^−6^ to 10^−4^ substitutions per nucleotide per round of copying (s/n/r) and the causes of this variability remain poorly understood. In addition to differences in intrinsic fidelity or error correction capability, viral mutation rates may be dependent on host factors. Here, we assessed the effect of the cellular environment on the rate of spontaneous mutation of the vesicular stomatitis virus (VSV), which has a broad host range and cell tropism. Luria-Delbrück fluctuation tests and sequencing showed that VSV mutated similarly in baby hamster kidney, murine embryonic fibroblasts, colon cancer, and neuroblastoma cells (approx. 10^−5^ s/n/r). Cell immortalization through p53 inactivation and oxygen levels (1–21%) did not have a significant impact on viral replication fidelity. This shows that previously published mutation rates can be considered reliable despite being based on a narrow and artificial set of laboratory conditions. Interestingly, we also found that VSV mutated approximately four times more slowly in various insect cells compared with mammalian cells. This may contribute to explaining the relatively slow evolution of VSV and other arthropod-borne viruses in nature.

## Author Summary

RNA viruses show high rates of spontaneous mutation, a feature that profoundly influences viral evolution, disease emergence, the appearance of drug resistances, and vaccine efficacy. However, RNA virus mutation rates vary substantially and the factors determining this variability remain poorly understood. Here, we investigated the effects of host factors on viral replication fidelity by measuring the viral mutation rate in different cell types and under various culturing conditions. To carry out these experiments we chose the vesicular stomatitis virus (VSV), an insect-transmitted mammalian RNA virus with an extremely wide cellular and host tropism. We found that the VSV replication machinery was robust to changes in cellular physiology driven by cell immortalization or shifts in temperature and oxygen levels. In contrast, VSV mutated significantly more slowly in insect cells than in mammalian cells, a finding may help us to understand why arthropod-borne viruses tend to evolve more slowly than directly transmitted viruses in nature.

## Introduction

RNA viruses show extremely high genetic variability and rapid evolution, ultimately due to their elevated rates of spontaneous mutation, which range from 10^−6^ to 10^−4^ substitutions per nucleotide per round of copying (s/n/r). However, mutation rate estimates vary considerably, even for the same virus [1], [2]. Since viral mutation rates have implications for pathogenesis [3], [4], vaccine development [5], [6] antiviral therapy [7], [8], and epidemiological disease management [9], [10], it is important to have accurate data and a clear understanding of the factors determining these rates. As a case in point, the risk of cross-species transmission is determined, in addition to the ecology of virus-host interactions, by the input of new adaptive mutations in the viral population [11], and a recent phylogenetic analysis of rabies virus isolates suggested that the waiting time required for host jumps depends on the number of positively selected mutations involved in cross-species transmission [12].

In RNA viruses, mutation rates are determined by the intrinsic base selection specificity of the viral polymerase [13]–[16], the presence/absence of proofreading mechanisms such as 3′exonuclease activity [17]–[19], or the mode of replication [20], [21]. However, in addition to these virus-encoded factors, viral mutation rates can be host-dependent. For instance, it has been suggested that the replicase of cucumber mosaic virus exhibits different fidelity in pepper and tobacco plants [22], [23]. In retroviruses, replication fidelity may be affected by intra-cellular dNTP imbalance and total concentration, which vary among cell types [24]–[26], although a recent study revealed no differences in the HIV-1 mutation rate in various cell types including T lymphoblast, glioblastoma and human embryonic kidney cells [27]. Also, the expression of host genes may influence the viral mutation rate as is the case of APOBEC3 cytidine deaminases, which can edit the HIV-1 cDNA and produce G-to-A hypermutations [28]–[30]. A similar role was postulated for the cellular RNA-dependent adenosine deaminase (ADAR) which could lead to A-to-G hypermutation in several RNA viruses, including rhabdoviruses [31], paramyxoviruses [32], and retroviruses [33]–[35]. Finally, cell metabolism may also have an impact in viral mutation rates, since it has been shown that ethanol-derived reactive oxygen species (ROS) can damage the RNA of hepatitis C virus, whereas other compounds such as glutathione and iron chelators were found to have the opposite effect [36].

Vesicular stomatitis virus (VSV) is a non-segmented negative-stranded RNA virus belonging to the family *Rhabdoviridae* with an extremely wide host tropism. The virion attaches to phosphadtidyl serine or other ubiquitous cell surface receptors and can productively infect most mammalian cells [37]. In nature, VSV infects a very large number of mammal species including livestock (cattle, horse, swine, goats, etc.) and wild animals (rodents, bear, lynx, bats, etc.), and also infects insects (sandflies, blackflies, mosquitoes, etc.) [38], [39], which act as transmission vectors [40]–[42]. Therefore, VSV replicates in widely different cellular environments, but the impact of this heterogeneity on the viral mutation rate is unknown. Actually, nearly all mutation rate estimates for animal viruses have been obtained in standard laboratory cell lines, which are usually immortalized or cancerous and thus show aberrant metabolic/mitotic rates and gene expression patterns. For VSV, most studies are conducted using hamster kidney cells, despite the fact that the brain is the main target organ of rhabdoviruses. Furthermore, all viral mutation rate studies have been conducted under atmospheric oxygen levels but these are substantially higher than those found in most tissues [43], and the impact of this type of environmental stress in the estimates is unknown. Here, we measured the mutation rate of VSV in primary and tumoral cell types including murine fibroblasts of various origins and neural cells, and under different oxygen levels, as well as insect cells. We found that the VSV mutation rate was relatively constant in all mammalian cells tested. However, VSV mutated four times more slowly in insect cells than in mammalian cells, a finding that may have implications for our understanding of arboviral evolution.

## Results/Discussion

### Fluctuation tests in BHK-21 cells

We measured the mutation rate of VSV by the Luria-Delbrück fluctuation test, a standard estimation method [44] that has been used previously in several viruses including poliovirus [45], vesicular stomatitis virus [46], influenza A virus [47], measles virus [48], turnip mosaic virus [49], and bacteriophages φ6 [20] and Qβ [50]. To score mutants, we used a monoclonal antibody against the envelope glycoprotein G and determined the probability of appearance of monoclonal antibody resistance (MAR) mutants in independent cultures (null-class method). First, we performed six independent tests in baby hamster kidney cells (BHK-21), for which we had previous results [46]. This gave an average mutation rate to the MAR phenotype of *m* = (1.64±0.27)×10^−5^ per round of copying (Table 1). This rate can be converted to per-nucleotide units as 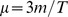, where *T* is the set of observable mutations leading to the phenotype (mutation target) and three stands for the number of possible nucleotide substitutions per site [2]. Sequencing of the glycoprotein G gene from 15 MAR plaques allowed us to identify four different nucleotide substitutions, which led to amino acid changes D257N, D259A, D259N, and S273T, whereas previous work reported the same substitutions at position 259 of the G glycoprotein in addition D257G, D257V, D257Y and A263E [51]. Taking *T* = 8, the estimated mutation rate is *μ* = 6.15×10^−6^ substitutions per nucleotide per round of copying (s/n/r).

**Table 1:** Fluctuation tests of VSV in BHK-21 cells.

	Test 1	Test 2	Test 3	Test 4	Test 5	Test 6
*N_i_* (pfu)	160±11	267±18	358±114	355±35	293±36	290±10
*N_f_* (pfu)	24562±1021	10875±956	20375±849	38150±1590	46157±1044	17200±934
Total cultures	24	24	24	24	24	24
With no MAR	16	20	17	16	16	15
With 1 MAR	6	4	6	7	7	4
With 2 MARs	0	0	1	1	0	2
With >2 MARs	2	0	0	0	1	3
Fraction with no MAR (*P*_0_)	0.667	0.833	0.708	0.667	0.667	0.625
Mutation rate (*m*)	1.66×10^−5^	1.71×10^−5^	1.72×10^−5^	1.07×10^−5^	0.88×10^−5^	2.78×10^−5^

### Validation by molecular clone sequencing

To verify the reliability of the above estimate, we used a molecular clone sequencing approach. This allowed us to score mutations more directly than in fluctuation tests and to analyze a wider genome region, although the interpretation of the data is complicated by the fact that the observed mutation frequency is dependent on selection, the number of generations elapsed, etc. BHK-21 cells were infected with a single infectious particle (i.e. plaque forming unit, pfu) by limiting dilution, and the resulting viral bursts (1.55×10^7^ final pfu on average) were used for RNA purification, RT-PCR, molecular cloning, and sequencing of three genome regions mapping to genes P, G, and L. We observed four single-nucleotide substitutions in 77500 bases in total, giving a mutation frequency of *f* = 5.16×10^−5^ (Table 2). For a per-cell burst size of *B* = 1250 [46], the number of infection cycles (i.e. viral generations) elapsed should be 
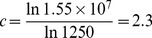
. Therefore, the per-generation increase in mutation frequency was 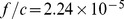. To account for the effect of selection, we used the previously characterized distribution of mutational fitness effects (see Methods). Based on this, the expected fraction of observable mutations after 2.3 generations was 53% and, thus, the estimated per-cell mutation rate is 
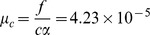
. The exact number of round of copying per cell is unknown but a previous work suggested *r_C_* = 5.8 rounds/cell, implying that *μ* = 7.30×10^−6^ s/n/r. This estimate is fully consistent with the results provided by the Luria-Delbrück fluctuation test. Subsequent experiments were done using fluctuation tests only because they provided a faster and simpler approach.

**Table 2:** Molecular clone sequencing of VSV from BHK-21 cells.

Gene	P	G	L
Genome sites	1339–1899	3858–4347	6974–7462
Clones sequenced	50	50	50
Total bases read	28050	25000	24450
Mutations	A1821C (Lys→Thr) G1640A (Gly→Arg)	A3983G (Glu→Glu) T3937A (Leu→His)	None

### Constant mutation rate in mammalian cells

**Figure 1:**
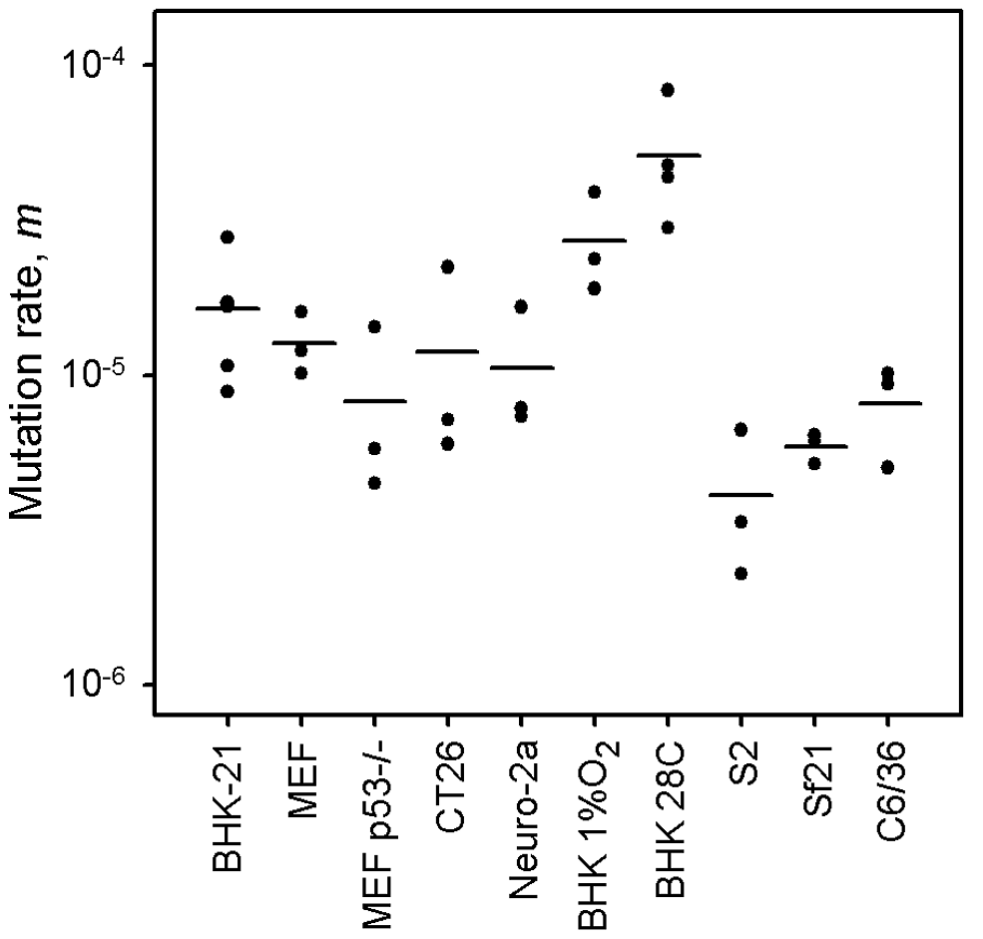
VSV mutation rate to the MAR phenotype estimated by the Luria-Delbrück fluctuation test in different cellular environments. Each dot represents an independent estimate (*n* = 3 for all except *n* = 6 for BHK-21 and *n* = 4 for BHK-21 at 28°C). Horizontal bars indicate the mean rate. Detailed information for each test is provided in Table 1 and in Text S1.

Previous mutation rate studies with VSV have been conducted in BHK-21 cells only [2], [46], [52]. However, these are immortalized/tumoral cells, as opposed to those typically encountered bythe virus in vivo. Furthermore, VSV has a tropism for neural cells, and kidney fibroblasts are not a natural target of the virus. Toaddress the potential effect of immortalization on the viral mutation rate, we performed fluctuation tests in primary mouse embryonic fibroblasts (MEFs) and isogenic, p53 knock-out, MEFs. The average rate was similar in normal (*m* = 1.27×10^−5^) and p53knock-out MEFs (*m* = 0.82×10^−5^), revealing no significant effectof cellular immortalization (Figure 1; t-test: *P* = 0.232, *n* = 6). However, many cell lines are tumoral and show other genetic andmetabolic alterations in addition to p53 inactivation. To check the potential effects of these changes, we performed fluctuation tests in CT26 cells from an undifferentiated grade IV colon adenocarcinoma of a BALB/c mouse [53], but we found no significant differences with primary MEFs (*m* = 1.18×10^−5^; t-test: *P* = 0.885, *n* = 6). Of note, BHK-21 are also tumor-forming cells, and the mutation rate was similar to the rate observed in MEFs or CT26 cells (one-way ANOVA: *P* = 0.293, *n* = 12). This homogeneity in the VSV mutation rate was not an obvious a priori, because metabolic and mitotic activity should alter the availability of NTPs [54] and hence could impact RNA replication fidelity, although VSV replicates in the cytoplasm and may not be strongly affected by these alterations. This result has implications for the field of oncolytic virotherapy [55], since it is critical to assess the genetic stability of these therapeutic viruses during large-scale manufacturing and clinical use. In particular, CT26 cells have been used in mice as a model for testing the oncolytic activity of VSV [56]. Also, the above results suggest that VSV replicates with similar fidelity in different cell types, but we sought to test whether this would also hold for neural cells. We therefore performed fluctuation tests in Neuro-2a cells from a mouse neuroblastoma [57]. Again, we found that the average mutation rate did not significantly differ from the rate obtained in BHK-21 cells (*m* = 1.06×10^−5^; t-test: *P* = 0.461, *n* = 9). Finally, to test for other potential effects of cell physiology, we also varied oxygen levels. The VSV mutation rate in BHK-21 cells cultured under hypoxic conditions (1% oxygen) was slightly higher but not significantly different to the rate obtained under standard conditions (*m* = 2.71×10^−5^; t-test: *P* = 0.122, *n* = 9). Oxidative stress should lead to the release of ROS, which have been previously shown to be mutagenic for hepatitis C virus [36]. However, VSV does not appear to be sensitive to oxidation levels. This might be related to the fact that the nucleocapsid of mononegavirales forms a tunnel-like structure which wraps the viral genomic RNA and remains assembled during the entire infection cycle [58], [59], effectively isolating the viral RNA [60].

### Lower mutation rate in insect cells

**Figure 2:**
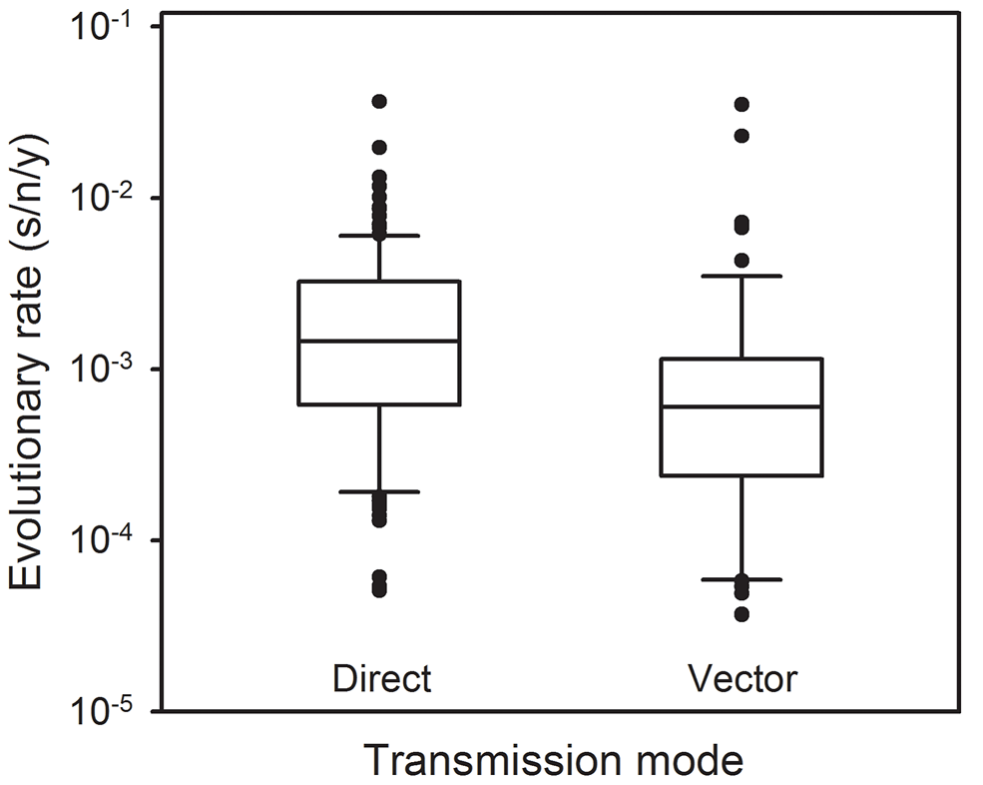
Molecular evolution of directly transmitted and arthropod-transmitted riboviruses. Data were collected from the supplementary information of a previous meta-analysis [69] and include 170 evolutionary rates, 113 for directly transmitted viruses and 57 for arboviruses. The box plot indicates the median (central lines), percentiles 25/75 (box) and percentiles 10/90 (bars), and outliers (dots).

Since VSV alternates between mammalian and insect hosts in nature, we sought to measure the viral mutation rate in insect cells (Figure 1). In S2 cells from *D. melanogaster* embryos, the average estimate from three independent fluctuation tests was *m* = 4.08×10^−6^, representing a fourfold decrease compared with BHK-21 (t-test: *P* = 0.009, *n* = 9). To further investigate this, we selected two additional insect cells lines: Sf21 ovarian cells from the moth *Spodoptera frugiperda*, and C6/36 from *Aedes albopictus* mosquito larvae. Also, since insect cells were infected at 28°C and mammalian cells at 37°C, we performed four additional tests in BHK-21 at 28°C. We used estimates obtained in mammalian (BHK-21, BHK-21 at 28°C, MEF, MEF p53−/−, CT26, and Neuro-2a) and insect cells (S2, Sf-21, and C6/36) to jointly test for the effects of host type and temperature (fixed factors) in a two-way ANOVA in which the specific cell line was treated as a random factor nested within host type. This confirmed that VSV shows lower mutation rate in insect cells than in mammalian cells (ANOVA: *P*<0.001), and also that temperature cannot account for this result because the estimates in BHK-21 were actually higher at 28°C than at 37°C (*P* = 0.001). Using log_10_-transformed data, the estimated effect size of the host type in the above model was 0.590±0.205, which implies a 3.9 fold mutation rate decrease in insect cells. One possible explanation for this difference is that our sensitivity to detect MAR mutants varied between assays performed in mammalian and insect cells. To address this, we first verified that MAR plating efficiency was similar in BHK-21, S2, Sf21, and C6/36 cells using a genetically engineered MAR mutant (D259A). Second, we tested for differences in the mutation target size (*T*). To do this, we sampled 15 individual MAR plaques from fluctuation tests performed in S2 cells and sequenced the region of the G protein controlling this phenotype. We found the same amino acid replacements as in fluctuation tests performed in BHK-21 cells (D257N, D259N, S273T, see above) except for D259A. However, because the D259 mutant is viable in insect cells [61], failure to detect it was probably due to insufficient sampling depth. We also found substitution A263E, which was reported previously in BHK-21 cells [51]. Therefore, insect S2 and BHK-21 cells shared a similar mutational repertoire and plating efficiency, supporting the consistency of the observed mutation rate difference. Interestingly, VSV [62] and arboviruses in general [63], [64] tend to evolve more slowly than directly transmitted viruses. Our own meta-analysis using 170 previously published evolutionary rates confirmed that, after accounting for phylogenetic relatedness and the timespan of sequence sampling, arboviruses showed a significantly lower evolution rate than directly transmitted viruses (Figure 2; two-way ANOVA: *P* = 0.006), the geometric mean rates being 5.7×10^−4^ substitutions per site per year (s/s/y) and 1.3×10^−3^ s/s/y, respectively. This has been often interpreted in terms of fitness tradeoffs, whereby neutral or beneficial mutations in mammals can be deleterious in insects, and vice versa, thus restricting viral evolution. However, whether arboviruses show similar mutation rates in mammalian and insect cells has not been addressed before, and our results offer a new possible explanation for the relatively slow arboviral evolution. Future experiments with other arboviruses could help elucidate the generality of these findings and, if so, to delineate the mechanisms behind the observed differences in replication fidelity.

## Materials and Methods

### Virus

Viruses were obtained from an infectious cDNA clone by transfecting baby hamster kidney (BHK-21) cells [65], [66], purified by filtration (0.22 µm), and stored at 70°C in aliquots until use.

### Cell culturing

BHK-21 cells (American Type Culture Collection, ATCC) were cultured in DMEM supplemented with 10% fetal bovine serum (FBS), 0.02 mM L-Glutamine, a mix of non-essential amino-acids, 100 µg/mL streptomycin, 60 µg/mL penicillin, and 2 µg/mL fungizone. MEFs and their p53−/− derivatives were obtained from Dr. Carmen Rivas (Centro Nacional de Biotecnología, Madrid) and cultured in the same medium but with 12% FBS. Neuro-2a cells were obtained from Prof. José M. García-Verdugo (Department of Cell Biology, University of Valencia) and cultured in MEM supplemented with 2 mM L-Glutamine, 1 mM sodium pyruvate, 10% FBS, non-essential amino acids and the above antibiotics. CT26 cells (ATCC) were cultured in DMEM with 10% FBS, 2 mM L-glutamine, 10 mM HEPES and antibiotics. All the above cells were incubated at 37°C with 5% C0_2_ and passaged upon confluence. *D. melanogaster* Schneider (S2) cells were obtained from Dr. Rubén Artero (Department of Genetics, University of Valencia) and cultured in Schneider's medium supplemented with 10% FBS and antibiotics at 25°C in the absence of C0_2_, and infected at 28°C. Sf21 cells were obtained from Dr. Salvador Herrero (Department of Genetics, University of Valencia) and were cultured in Grace's insect medium supplemented with 10% FBS and antibiotics at 28°C in the absence of C0_2_. C6/36 cells (ATCC) were cultured in DMEM supplemented with 10% FBS, 2 mM L-glutamine, non-essential amino acids, 1 mM sodium pyruvate and antibiotics at 28°C under 5% C0_2_. Hypoxia was achieved by displacing oxygen with nitrogen, using a Galaxy 170R incubator (Eppendorf).

### Luria-Delbrück fluctuation tests

We inoculated 32 identical cultures each containing 10^4^ confluent cells with approximately 300 pfu/well (*N*_i_) and incubated them until approximately 3×10^4^ pfu/well were produced (*N*_f_). After a round of freeze-thawing to release intracellular particles, we used eight cultures for titration and 24 for plating the entire undiluted volume (100 µL) in the presence of a monoclonal antibody against the surface glycoprotein G at a concentration that neutralizes completely the wild-type virus and selects for MAR mutants. The antibody, in the form of a hybridoma supernatant, was added to the plating medium (25% v∶v) to avoid phenotypic masking [52]. Plating assays were done in DMEM gelled with 0.4% agarose containing 2% FBS. After 24 h, monolayers were fixed with 10% formaldehyde and stained with 2% crystal violet to visualize plaques. Since mutation is a rare event, the number of mutations per culture is expected to follow a Poisson distribution of parameter 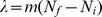 and therefore the probability of observing no mutants in a culture is 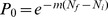, where *m* is the mutation rate from the wild-type to the MAR phenotype (null-class method). However, if there is incomplete plating, some cultures may contain undetected MAR mutants. If we define *z* as the plating efficiency (relative to BHK-21 cells), the probability of observing no mutants can be expressed as 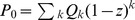, where 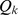 is the probability of *k* actual mutants in a culture. Using a Poisson distribution of parameter 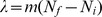 for *k*, we numerically solved *Q*_0_ given *P*_0_, *N*_i_, *N*_f_, and *z* and calculated the mutation rate as 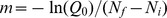.

### Plating efficiency in fluctuation tests

For each cell type tested, the plaque assay for scoring MAR mutants was done in BHK-21 cells for technical feasibility and to control for differences in plating efficiency among cells. However, since plaque assays to score MAR mutants were done without dilution, antiviral cytokines or other compounds released from the cells in which the virus was grown could modify plating efficiency (plaque assays for determining *N*_f_ were done at a roughly 1/100 dilution and thus were much less affected by this problem). For instance, BHK-21 cells are at least partially responsive to interferon [67], potentially inhibiting growth of MAR mutants and biasing mutant counts down. To calibrate this effect, we titrated a MAR clone obtained by site-directed mutagenesis (substitution D259A in the surface glycoprotein G) in the presence of undiluted supernatants harvested from cells previously infected with the wild-type virus (*N*_i_≈300 pfu and *N*_f_>≈10^4^ pfu, similar to fluctuation tests), adding monoclonal antibody to the plates to observe MAR plaques only. The wild-type infections were performed under each of the experimental conditions (BHK-21, MEF, MEF p53−/−, CT26, Neuro-2a, BHK-21 with 1% O_2_, BHK-21 at 28°C, S2, Sf21 and C6/36 cells). Addition of supernatants from BHK-21 cells infected under standard conditions did not alter the titer of the D259 MAR clone, hence the relative plating efficiency was *z* = 1. The relative plating efficiency for each of the other conditions is shown in the Supporting Information “Text S1” and was based on at least six independent plating assays.

### Mutation target size in fluctuation tests

To ascertain the number of possible mutations conferring the MAR phenotype, we plated approximately 10^5^ pfu in the presence of antibody, incubated them for 24 h, and pipetted individual plaques. Viral RNA was purified, reverse-transcribed using AccuScript High Fidelity Reverse Transcripatse (Agilent Technologies), and the cDNA was PCR-amplified using Phusion High Fidelity DNA polymerase (New England Biolabs). We used specific primers to amplify and sequence a region of the G protein (genome sites 3361 to 4501 in GenBank accession EF197793) which controls the MAR phenotype [51]. PCR products were sequenced by the Sanger method and analyzed using Staden software.

### Mutation frequency determination by molecular clone sequencing

A 96-well plate containing 10^4^ cells per well was inoculated with a limiting dilution of the viral stock such that approximately 10% of wells were infected. Plates were incubated at 37°C for 24 h, inspected under the microscope for cytopathic effects, and freeze-thawed to allow release of intracellular viruses. Viral RNA was purified from the supernatant of each of five positive wells and reverse-transcribed using AccuScript High Fidelity Reverse Transcripatse, and the cDNA was PCR-amplified using Phusion High Fidelity DNA polymerase and specific primers located in the P, G and L genes, as indicated. PCR products were cloned and used for *E.coli* transformation, and 10 colonies were picked and amplified by colony PCR using Phusion High Fidelity DNA polymerase. PCR products were sequenced by the Sanger method and analyzed using Staden software. To obtain the mutation frequency, the number of observed mutations was divided by the total number of bases sequenced.

### Effect of selection on mutation frequency

We used the empirically characterized distribution of mutational fitness effects of random single-nucleotide substitutions in VSV to correct for the effect of selection on mutation frequency and obtain the mutation rate per cell infection. We did so numerically by simulating the combined effects of mutation and selection. The statistical distribution of fitness effects (*s*) for viable substitutions can be roughly captured using an exponential distribution truncated at 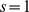 (lethality) plus a class of lethals occurring with probability *p_L_*: 
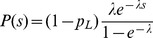
 if 0<*s*<1, 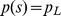 if *s* = 1, and 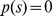 otherwise. In a previous work using the same VSV strain as here, it was estimated that 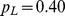 and that 
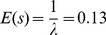
 [65], [68]. Fitness effects were measured as growth rate ratios, 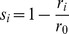, where *r* is the exponential growth rate and subscripts *i* and *0* refer to the mutant and wild-type, respectively [65]. These *s*-values were transformed to per cell infection units as 
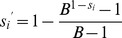
, where *B* is the burst size. After simulating fitness effects using the truncated exponential plus lethal distribution and applying the per cell infection transformation, selection was applied by picking individuals for the next cell infection cycle with weighted probability 1−s′, and the process was iterated. This provided an expected mutation frequency *f* and therefore a relationship between *μ* and *f*. Genetic drift was ignored since it should not modify the expected value of *f*. Also, for simplicity, mutations were assumed to have independent fitness effects (no epistasis) and back mutations were ignored, which seems reasonable in the short-term, because single forward mutations will greatly outnumber secondary and back mutations. Simulations were performed using Wolfram Mathematica and Excel. A graphical representation of this correction can be found in a previous work [2].

### Analysis of published molecular evolutionary rates

In a previous meta-analysis, we collected evolutionary rate estimates that were originally inferred from field isolates using Bayesian analysis of dated sequences after validation of the molecular clock [69]. Here, we used 170 of these estimates, which corresponded to 62 different riboviruses. We sought to compare viruses transmitted directly through respiratory secretions, blood, sexual contact, feces, or animal bites (n = 113) against arboviruses (n = 57). We used a two-way ANOVA in which the following factors were included: transmission mode (fixed), viral family (random) to account for phylogenetic relatedness, and sampling timespan (covariate) to account for the known time-dependency of evolution rate estimates. Since rates ranged several orders of magnitude log-transformed data were used.

## Supporting Information

Text S1 Detailed information of fluctuation test results for wild-type MEFs (Table S1), p53−/− MEFs (Table S2), CT26 colon cancer cells (Table S3), Neuro-2a neuroblastoma cells (Table S4), BHK-21 cells under hypoxia (Table S5), BHK-21 cells at 28°C (Table S6), S2 cells (Table S7), in Sf21 cells (Table S8), and C6/36 cells (Table S9).
(PDF)
