# Supplementary material for: Variation in RNA Virus Mutation Rates across Host Cells
Source: PLoS Pathog. 2014 Jan 23;10(1):e1003855. doi: 10.1371/journal.ppat.1003855 (PMC3900646; doi:10.1371/journal.ppat.1003855)
Supplement: Text S1 — Detailed information of fluctuation test results for wild-type MEFs (Table S1), p53−/− MEFs (Table S2), CT26 colon cancer cells (Table S3), Neuro-2a neuroblastoma cells (Table S4), BHK-21 cells under hypoxia (Table S5), BHK-21 cells at 28°C (Table S6), S2 cells (Table S7), in Sf21 cells (Table S8), and C6/36 cells (Table S9). (PDF) [file ppat.1003855.s001.pdf]

Supplementary Table S1: fluctuation tests in wild-type MEFs.

| | **Test 1** | **Test 2** | **Test 3** |
|---|---|---|---|
| $N_i$ (pfu) | 439 ± 94 | 124 ± 15 | 160 ± 11 |
| $N_f$ (pfu) | 49812 ± 5985 | 12000 ± 2021 | 36075 ± 3162 |
| Total cultures | 24 | 24 | 24 |
| With no MAR | 15 | 21 | 14 |
| With 1 MAR | 5 | 3 | 10 |
| With 2 MARs | 2 | 0 | 0 |
| With >2 MARs | 2 | 0 | 0 |
| Fraction no MAR ($P_0$) | 0.625 | 0.875 | 0.583 |
| Plating efficiency ($z$)[a] | 0.94 ± 0.04 | 0.94 ± 0.04 | 0.94 ± 0.04 |
| Corrected $P_0$ ($Q_0$) | 0.607 | 0.868 | 0.564 |
| Mutation rate ($m$) | $1.01 \times 10^{-5}$ | $1.20 \times 10^{-5}$ | $1.60 \times 10^{-5}$ |

a: relative to fluctuation tests performed in BHK-21 cells.

Supplementary Table S2: fluctuation tests in p53-/- MEFs.

| | **Test 1** | **Test 2** | **Test 3** |
|---|---|---|---|
| $N_i$ (pfu) | 33 ± 10 | 368 ± 38 | 160 ± 11 |
| $N_f$ (pfu) | 40929 ± 6820 | 9725 ± 1619 | 40650 ± 2602 |
| Total cultures | 24 | 24 | 24 |
| With no MAR | 20 | 21 | 19 |
| With 1 MAR | 4 | 2 | 3 |
| With 2 MARs | 0 | 1 | 2 |
| With >2 MARs | 0 | 0 | 0 |
| Fraction no MAR ($P_0$) | 0.833 | 0.875 | 0.792 |
| Plating efficiency ($z$)[a] | 1.01 ± 0.02 | 1.01 ± 0.02 | 1.01 ± 0.02 |
| Corrected $P_0$ ($Q_0$) | 0.833 | 0.875 | 0.792 |
| Mutation rate ($m$) | $4.46\times 10^{-6}$ | $1.43 \times 10^{-5}$ | $5.77 \times 10^{-6}$ |

a: relative to fluctuation tests performed in BHK-21 cells ($z$ = 1 was used).

Supplementary Table S3: fluctuation tests in CT26 colon cancer cells.

| | **Test 1** | **Test 2** | **Test 3** |
|---|---|---|---|
| $N_i$ (pfu) | 465 ± 52 | 33 ± 10 | 123 ± 15 |
| $N_f$ (pfu) | 42375 ± 5214 | 6675 ± 1217 | 134700 ± 4435 |
| Total cultures | 24 | 24 | 24 |
| With no MAR | 22 | 23 | 19 |
| With 1 MAR | 2 | 1 | 3 |
| With 2 MARs | 0 | 0 | 1 |
| With >2 MARs | 0 | 0 | 1 |
| Fraction no MAR ($P_0$) | 0.917 | 0.958 | 0.792 |
| Plating efficiency ($z$)[a] | 0.29 ± 0.05 | 0.29 ± 0.05 | 0.29 ± 0.05 |
| Corrected $P_0$ ($Q_0$) | 0.741 | 0.863 | 0.447 |
| Mutation rate ($m$) | $7.16 \times 10^{-6}$ | $2.23 \times 10^{-5}$ | $5.99 \times 10^{-6}$ |

a: relative to fluctuation tests performed in BHK-21 cells.

Supplementary Table S4: fluctuation tests in Neuro-2a neuroblastoma cells.

| | **Test 1** | **Test 2** | **Test 3** |
|---|---|---|---|
| $N_i$ (pfu) | 124 ± 15 | 160 ± 11 | 215 ± 36 |
| $N_f$ (pfu) | 28913 ± 434 | 61275 ± 3417 | 29513 ± 3244 |
| Total cultures | 24 | 24 | 24 |
| With no MAR | 16 | 16 | 20 |
| With 1 MAR | 3 | 2 | 4 |
| With 2 MARs | 1 | 6 | 0 |
| With >2 MARs | 4 | 0 | 0 |
| Fraction no MAR ($P_0$) | 0.667 | 0.667 | 0.833 |
| Plating efficiency ($z$)[a] | 0.85 ± 0.06 | 0.85 ± 0.06 | 0.85 ± 0.06 |
| Corrected $P_0$ ($Q_0$) | 0.621 | 0.621 | 0.807 |
| Mutation rate ($m$) | $1.65\times 10^{-5}$ | $7.80 \times 10^{-6}$ | $7.34 \times 10^{-6}$ |

a: relative to fluctuation tests performed in BHK-21 cells.

Supplementary Table S5: fluctuation tests in BHK-21 cells under hypoxia (1% $O_2$).

| | **Test 1** | **Test 2** | **Test 3**[b] |
|---|---|---|---|
| $N_i$ (pfu) | 355 ± 35 | 267 ± 18 | 267 ± 18 |
| $N_f$ (pfu) | 49875 ± 175 | 19625 ± 1735 | 20750 ± 1652 |
| Total cultures | 24 | 24 | 24 |
| With no MAR | 15 | 19 | 16 |
| With 1 MAR | 4 | 4 | 6 |
| With 2 MARs | 3 | 0 | 2 |
| With >2 MARs | 2 | 1 | 0 |
| Fraction no MAR ($P_0$) | 0.625 | 0.792 | 0.667 |
| Plating efficiency ($z$)[a] | 0.51± 0.04 | 0.51± 0.04 | 0.51± 0.04 |
| Corrected $P_0$ ($Q_0$) | 0.398 | 0.633 | 0.452 |
| Mutation rate ($m$) | $1.90 \times 10^{-5}$ | $2.36 \times 10^{-5}$ | $3.88 \times 10^{-5}$ |

a: relative to fluctuation tests performed in BHK-21 cells.

b: performed the same day and from the same viral stock as test 2.

Supplementary Table S6: fluctuation tests in BHK-21 cells at 28°C.

| | **Test 1** | **Test 2**[b] | **Test 3**[b] | **Test 4** |
|---|---|---|---|---|
| $N_i$ (pfu) | 327 ± 11 | 327 ± 11 | 327 ± 11 | 295 ± 12 |
| $N_f$ (pfu) | 17667 ± 3781 | 13967± 4151 | 4900 ± 1052 | 4600 ± 708 |
| Total cultures | 24 | 24 | 24 | 24 |
| With no MAR | 15 | 14 | 17 | 20 |
| With 1 MAR | 3 | 2 | 5 | 4 |
| With 2 MARs | 3 | 4 | 0 | 0 |
| With >2 MARs | 3 | 4 | 2 | 0 |
| Fraction no MAR ($P_0$) | 0.625 | 0.583 | 0.708 | 0.833 |
| Plating efficiency ($z$)[a] | 0.91± 0.05 | 0.91± 0.05 | 0.91± 0.05 | 0.91± 0.05 |
| Corrected $P_0$ ($Q_0$) | 0.597 | 0.553 | 0.685 | 0.815 |
| Mutation rate ($m$) | $2.98 \times 10^{-5}$ | $4.35 \times 10^{-5}$ | $8.29 \times 10^{-5}$ | $4.76 \times 10^{-5}$ |

a: relative to fluctuation tests performed in BHK-21 cells.

b: performed the same day and from the same stock as test 1.

Supplementary Table S7: fluctuation tests in S2 cells.

| | **Test 1** | **Test 2** | **Test 3** |
|---|---|---|---|
| $N_i$ (pfu) | 358 ± 11 | 358± 11 | 322 ± 10 |
| $N_f$ (pfu) | 19025 ± 898 | 6775 ± 724 | 13080 ± 593 |
| Total cultures | 24 | 24 | 24 |
| With no MAR | 23 | 23 | 23 |
| With 1 MAR | 1 | 1 | 0 |
| With 2 MARs | 0 | 0 | 0 |
| With >2 MARs | 0 | 0 | 1 |
| Fraction with no MAR ($P_0$) | 0.958 | 0.958 | 0.958 |
| Plating efficiency ($z$)[a] | 1.07 ± 0.07 | 1.07 ± 0.07 | 1.07 ± 0.07 |
| Corrected $P_0$ ($Q_0$) | 0.958 | 0.958 | 0.958 |
| Mutation rate ($m$) | $2.28 \times 10^{-6}$ | $6.63 \times 10^{-6}$ | $3.34 \times 10^{-6}$ |

a: relative to fluctuation tests performed in BHK-21 cells.

Supplementary Table S8: fluctuation tests in Sf21 cells.

| | **Test 1** | **Test 2** | **Test 3** |
|---|---|---|---|
| $N_i$ (pfu) | 295 ± 12 | 223 ± 26 | 312 ± 32 |
| $N_f$ (pfu) | 6970 ± 174 | 14400 ± 898 | 17033 ± 652 |
| Total cultures | 24 | 24 | 24 |
| With no MAR | 23 | 22 | 22 |
| With 1 MAR | 1 | 2 | 1 |
| With 2 MARs | 0 | 0 | 1 |
| With >2 MARs | 0 | 0 | 0 |
| Fraction with no MAR ($P_0$) | 0.958 | 0.917 | 0.917 |
| Plating efficiency ($z$)[a] | 1.09 ± 0.04 | 1.09 ± 0.04 | 1.09 ± 0.04 |
| Corrected $P_0$ ($Q_0$) | 0.958 | 0.917 | 0.917 |
| Mutation rate ($m$) | $6.38 \times 10^{-6}$ | $6.14 \times 10^{-6}$ | $5.20 \times 10^{-6}$ |

a: relative to fluctuation tests performed in BHK-21 cells.

Supplementary Table S9: fluctuation tests in C6/36 cells.

| | **Test 1** | **Test 2** | **Test 3** |
|---|---|---|---|
| $N_i$ (pfu) | 295 ± 12 | 223 ± 26 | 312 ± 32 |
| $N_f$ (pfu) | 10100 ± 410 | 9800 ± 546 | 10833 ± 499 |
| Total cultures | 24 | 24 | 24 |
| With no MAR | 22 | 23 | 22 |
| With 1 MAR | 1 | 0 | 2 |
| With 2 MARs | 1 | 1 | 0 |
| With >2 MARs | 0 | 0 | 0 |
| Fraction with no MAR ($P_0$) | 0.916 | 0.958 | 0.916 |
| Plating efficiency ($z$)[a] | 0.88 ± 0.02 | 0.88 ± 0.02 | 0.88 ± 0.02 |
| Corrected $P_0$ ($Q_0$) | 0.906 | 0.953 | 0.906 |
| Mutation rate ($m$) | $1.01 \times 10^{-5}$ | $5.01 \times 10^{-6}$ | $9.32 \times 10^{-6}$ |

a: relative to fluctuation tests performed in BHK-21 cells.
